# Microbiological and Clinical Short-Term Evaluation of the Efficacy of an Herbal Tincture as an Adjunctive Treatment in the Management of Stage II, Grade A Periodontitis

**DOI:** 10.3390/pathogens14090861

**Published:** 2025-08-29

**Authors:** Milica Petrović, Ljiljana Kesić, Bojana Miladinović, Radmila Obradović, Ana Pejčić, Marija Bojović, Katarina Šavikin, Jelena Živković, Ivana Stanković, Dušanka Kitić

**Affiliations:** 1 Department of Oral Medicine and Periodontology, Dental Clinic, Faculty of Medicine, University of Niš, 18108 Niš, Serbia; ljiljana.kesic@medfak.ni.ac.rs (L.K.); radmila.obradovic@medfak.ni.ac.rs (R.O.); ana.pejcic@medfak.ni.ac.rs (A.P.); marija.bojovic@medfak.ni.ac.rs (M.B.); ivana.stankovic.stomatologija@medfak.ni.ac.rs (I.S.); 2 Department of Pharmacy, Faculty of Medicine, University of Niš, 18108 Niš, Serbia; bojana.miladinovic@medfak.ni.ac.rs; 3 Institute for Medicinal Plants Research “Dr. Josif Pančić”, Tadeuša Košćuška 1, 11000 Belgrade, Serbia; ksavikin@mocbilja.rs (K.Š.); jzivkovic@mocbilja.rs (J.Ž.)

**Keywords:** periodontal disease, anaerobic bacteria, herbal remedies, polymerase chain reaction

## Abstract

The increased incidence of periodontitis, the resistance of periodontal pathogens to antibiotics, and the adverse effects of certain drugs used in general dentistry present a strong rationale for seeking safe and effective plant-based treatments for periodontitis. HPLC-DAD analysis of a commercial herbal tincture confirmed the presence of rosmarinic acid (1102.79 ± 21.56 µg/mL), luteolin-7-*O*-glucoside (358.06 ± 5.64 µg/mL), and isorhamnetin (24.17 ± 0.49 µg/mL), bioactive phytochemicals known for their antimicrobial and anti-inflammatoryproperties. The randomized prospective study analyzed Tinctura paradentoica^®^ as an adjunct to anti-infectious non-surgical periodontal therapy (NSPT) on clinical and microbiological parameters in patients with moderate periodontitis (Stage II, Grade A). All 60 recruited participants were randomly allocated to either the intervention group (NSPT + Tinctura paradentoica^®^) or the control group (NSPT alone). The rate of prevalence of the following periodontopathogenic microorganisms (*Treponema denticola*, *Tannerella forsythensis*), assessed by polymerase chain reaction (PCR) analysis, was significantly lower in the intervention group (*p* < 0.001), but no statistically significant difference was found for *Porphyromonas gingivalis*. The herbal tincture, combined with NSPT, produces a short-term reduction in periodontal clinical parameters (Green–Vermilion plaque index, bleeding on probing index (BOP), and clinical attachment level (CAL), without clinical relevance, and the prevalence of the following bacteria species (*Tannerella forsythensis*, *Treponema denticola*).

## 1. Introduction

Periodontitis is a chronic inflammatory condition involving the immune-mediated destruction of tooth-supporting tissues in response to microbial biofilms [1]. Advances in molecular techniques and high-throughput technologies have enhanced understanding of the complexity of the periodontal microbiome [2]. It affects approximately 20–50% of the global population and is clinically manifested by gingival inflammation, clinical attachment loss (CAL), radiographically evident alveolar bone resorption, periodontal pocket formation, bleeding on probing, increased tooth mobility, and tooth exfoliation in advanced stages [3]. Although dysbiosis of the local microbial association triggers local inflammation, over-activation of the host immune response directly activates osteoclastic activity and alveolar bone loss [4]. The primary pathogens of periodontitis have been identified in bacteria, belonging to the so-called “red complex” (*Porphyromonas gingivalis*, *Tannerella forsythensis*, and *Treponema denticola*), which is considered the leading cause of changes in polymicrobial communities, while *Aggregatibacter actinomycetemcomitans* plays a key role in the development of formerly classified aggressive periodontitis, by stimulating macrophages to produce proinflammatory cytokines, including interleukin-1 (IL-1), IL-1β and tumor necrosis factor (TNF) [5,6].

Phytotherapy is increasingly acknowledged in dentistry for offering natural agents with better biocompatibility, reduced toxicity, and clinically validated therapeutic effects, often surpassing those of traditional medications [7]. Natural products have been used as a significant source of substances for controlling oral diseases, especially in managing plaque-related diseases, such as periodontal disease. Consequently, the phytopreparation Tinctura paradentoica^®^ was selected for evaluation in this study. This tincture contains marigold tincture (Tinct. *Calendulae*), yarrow tincture (Tinct. *Millefolii*), oregano tincture (Tinct. *Origani*), knotgrass tincture (Tinct. *Polygoni*), sage tincture (Tinct. *Salviae*), tormentil tincture (Tinct. *Tormentillae*), and peppermint essential oil (*Aetheroleum Menthae piperitae*). Marigold (*Calendula officinalis* L., Asteraceae) has demonstrated anti-inflammatory and anti-oxidant activities. Tinctura paradentoica^®^, a hydroalcoholic extract derived from medicinal plant material, has been traditionally used for managing inflammation and infections in the oral cavity. However, despite its use, scientific data supporting its efficacy remain limited, particularly regarding its chemical composition and mechanism of action. Understanding the polyphenolic profile of the tincture is essential to understanding the pharmacological basis of its efficacy and to support its standardization and quality control. In this context, high-performance liquid chromatography coupled with diode-array detection (HPLC-DAD) provides a robust and reliable analytical method for the qualitative and quantitative assessment of phenolic constituents.

In periodontal diseases, used as a gel or mouthwash, marigold extracts have shown positive effects on gingivitis and mild periodontitis treatment due to the presence of phenolic compounds-flavonols, mostly quercetin [8,9,10]. Yarrow (*Achillea millefolium* L., Asteraceae) has been used for centuries in traditional medicine, among other things, due to its anti-inflammatory effect. The presence of phenolic acids (chlorogenic and dicaffeoylquinic acids), flavonoids (luteolin, apigenin, and quercetin), and essential oils has been related to antioxidant, anti-inflammatory, and antibacterial properties [11]. Yarrow ethanolic extracts suppressed the production of pro-inflammatory cytokines [12,13]. Ethanol extracts of oregano (*Origanum vulgare* L., Lamiaceae) have been noted as a good source of compounds with antimicrobial, anti-biofilm, and antiviral activity against dental plaque bacteria [14]. Flavonoids of knotgrass (*Polygonum aviculare* L., Polygonaceae) are presumed to be responsible for their anti-inflammatory activity [15]. Due to its anti-inflammatory properties, sage (*Salvia officinalis* L., Lamiaceae) has been recommended for treating pharyngitis, stomatitis, gingivitis, and periodontitis [16]. Sage extract is used as a component in several different mouthwashes [17]. Tormentil (*Potentilla erecta* (L.) Raeusch, Rosaceae) has been used in traditional medicine for the symptomatic treatment of mild inflammation of the oral and pharyngeal mucosa. These effects are mainly due to polyphenols and tannins [18,19]. Peppermint (*Mentha x piperita* L., Lamiaceae) contains an essential oil with an analgesic property [20]. Essential oils and mint leaves are used to make mouthwashes and gels that act against periodontopathogenic bacteria [21]. Tannins, flavonoids and essential oils dominate the composition of these tinctures. This phytopreparation has astringent, antiseptic, anti-inflammatory, mild anesthetic, and hemostatic effects thanks to its complex composition.

In the context of maintaining periodontal health in previously treated patients, there is scarce evidence supporting the effectiveness of alternative or supplementary approaches alongside standard mechanical plaque removal. Trombelli et al. suggest that in patients undergoing supportive periodontal therapy every 3–4 months, using alternative methods like Er:YAG laser or adjuncts such as subantimicrobial-dose doxycycline or photodynamic therapy does not provide additional clinical improvements in preventing further attachment loss compared to mechanical debridement alone [22]. While Suvan et al. analyzed data from randomized controlled trials with a six-month follow-up, their findings support the effectiveness of mechanical subgingival debridement in the non-surgical treatment of periodontitis, regardless of the type of instrument used or the method of application [23].

The current study aimed to evaluate the short-term effects (one-month) of phytopreparation as an additional treatment alongside NSPT by assessing its impact on clinical periodontal parameters and anaerobic bacteria from the “red complex” (*Porphyromonas gingivalis*, *Tannerella forsythensis*, and *Treponema denticola*) in patients with moderate periodontitis (stage II, grade A).

## 2. Materials and Methods

### 2.1. Study Design

The randomized prospective study included 60 subjects, who signed an informed consent form. Neither the investigator nor the participant was aware of the assignment before agreeing to participate in the investigation. Patients were divided into two groups: the control group included 30 patients with moderate periodontitis, who received non-surgical periodontal treatment (NSPT), and the intervention group included 30 patients with moderate periodontitis (Stage II, Grade A), who received phytotherapy after NSPT. Randomization was performed using a computer-generated sequence in blocks of equal size to ensure balanced allocation between groups. Allocation concealment was maintained through the use of sequentially numbered, opaque, sealed containers prepared by an independent examiner not involved in patient recruitment or outcome assessment. Participants were blinded to group assignment, as neither they nor the investigators responsible for recruitment were aware of allocation prior to enrollment. All clinical examinations were performed by a single calibrated examiner who was blinded to the treatment allocation. Laboratory personnel conducting the microbiological analyses processed all samples under coded identifiers and were not informed of the participants’ group assignments.

All patients received oral hygiene instructions and non-surgical periodontal treatment (removal of dental biofilm by polishing paste, followed by scaling and root planning by ultrasonic scaler Woodpecker (UDS-J, Medical Instrument Company, Guilin, Guangxi, China) and curettage of periodontal pockets (with Gracey 5–6, hand and 2R-2L Columbia universal curettes Hu Friedy, Chicago, IL, USA) for five consecutive days by one periodontist.

Afterwards, in the intervention group, 30 patients with moderate periodontitis received phytotherapy after non-surgical periodontal treatment for five consecutive days. The phytotherapy was carried out by using a solution of Tinctura paradentoica^®^ (Institute “Dr Josif Pancic”, Beograd, Republic of Serbia). The solution was applied using a 23-gauge needle, angled at approximately 110° along the length of its shaft, and a sterile single-use syringe (5 mL) in a volume of 0.1 mL per periodontal pocket (subgingival irrigation of all the pockets, 2 min per quadrant, isolated with cotton rolls placement). The applied solution contains 15% tincture of calendula, 20% millefolium flower tincture, 20% oregano tincture, 20% tincture *Polygoni*, 20% tincture salvia, 5% Tormentillae rhizome tincture (tinct.) (Tinct. *Tormentillae*) and 0.02% essential oil from mint (*Aetheroleum Menthae piperitae*).

### 2.2. High-Performance Liquid Chromatography (HPLC) of Tinctura Paradentoica^®^

Polyphenolic compounds in Tinctura paradentoica were analyzed using a high-performance liquid chromatography (HPLC) system (Agilent 1260 RR, Agilent Technologies, Waldbronn, Germany) equipped with a diode-array detector (DAD) scanning from 190 to 550 nm. Chromatographic separation was achieved on a Zorbax SB-C18 reversed-phase column (150 mm × 4.6 mm, 5 μm particle size, Agilent). The mobile phases consisted of (A) 1% (*v*/*v*) orthophosphoric acid in water and (B) acetonitrile. The gradient elution program was as follows: 0–2.6 min, 90–85% A; 2.6–8 min, 85% A; 8–10.8 min, 85–80% A; 10.8–18 min, 80% A; 18–23 min, 80–70% A; 23–25 min, 70–50% A; 25–27 min, 50–30% A; 27–29 min, 30–10% A; 29–31 min, 10–0% A; and 31–34 min, 0% A. The DAD was set to monitor absorbance at 260, 280, 320, and 360 nm. The column was maintained at 40 °C, with a flow rate of 0.8 mL/min and an injection volume of 8 μL. Identification of the compounds was based on comparisons of their retention times and UV spectra with those of known reference standards. Quantification was carried out using calibration curves, and the results were reported as micrograms per gram of dry weight (μg/g DW).

### 2.3. Ethical Approval

The Ethical Committee of the Clinic of Dentistry, Medical Faculty, University of Niš approved the realization of research (No 20/3-2019-8 EO), which was written in accordance with the principles of the Helsinki Declaration.

### 2.4. Inclusion Criteria

Patients were eligible for inclusion if they fulfilled the following diagnostic criteria for moderate periodontitis (Stage II, Grade A) according to the 2018 classification scheme for periodontal and peri-implant diseases and conditions [24]: clinical attachment loss (CAL) of 3–4 mm, radiographic evidence of bone loss ranging from 15% to 33%, predominantly horizontal in pattern, no history of tooth loss attributable to periodontitis, and the presence of both supragingival and subgingival hard deposits. The extent of periodontal destruction was assessed using orthopantomography.

### 2.5. Exclusion Criteria

Patients who had undergone any surgical/non-surgical periodontal therapy or antibiotic treatment in the past 6 months; who had acute or chronic infections, systemic illness, autoimmune diseases, hemorrhagic disorders, allergy, or hypersensitivity to any active or inactive component of the phytopreparation; or patients who had undergone chemo- and/or radiotherapy; as well as pregnant and lactating women and smokers were excluded from the study.

### 2.6. Sample Size Calculation

The sample size calculation determined that the minimum number of subjects was 7 per treatment group, based on a previously published study [25] and required parameters: power of study 0.8 and α error probability 0.05 (significance level), using the change in CAL as a primary outcome variable. While the calculated sample size is small, increasing the sample size to 30 participants is advisable because some of them may be lost during the study.

### 2.7. Clinical Examinations

The calibration training was performed before the beginning of the study. One periodontist (examiner) underwent the activity that lasted two days, where his reproducibility in duplicating measurements of 10 patients with moderate periodontitis (not involved in the study) was within a 1 mm difference in 96% of the recordings.

At the baseline examination and the re-examinations (the 5-day session treatments and the 30 days post-therapy), the Green-Vermilion plaque index was recorded: mesial, distal, buccal, and lingual surfaces were assessed as follows: 0 = no plaque present; 1 = plaque covering not more than 1/3 of the tooth; 2 = plaque covering more than 1/3 but not more than 2/3 of exposed tooth surface; and 3 = plaque covering more than 2/3 of exposed tooth surface. Gingival bleeding on probing index (BOP) was evaluated using a scoring scheme: 0 = no bleeding within 10 s after probing; 1 = bleeding within 10 to 20 s after probing; and 2 = bleeding on probing. Clinical attachment level (CAL) is the distance between the periodontal pocket base and the cemento-enamel junction (defined in millimeter (mm) score), performed by manual probe (probing pressure, 0.25 N).

### 2.8. Sampling and Polymerase Chain Reaction (PCR) Analysis

Subgingival samples were collected at baseline examination and after the fifth treatment to quantify the following bacteria: *Porphyromonas gingivalis*, *Tannerella forsythensis*, and *Treponema denticola* by PCR analysis. The PCR procedure was conducted by absorbent paper points (DiaDent Group International Inc., Chongju, Republic of Korea) placed in the deepest approximal periodontal pocket in each dental quadrant. The samples were placed in sterile Eppendorf tubes collected from the deepest pocket in each quadrant (60 patients, 6 sites, 4 paper points for each site) at baseline and after treatment using absorbent paper points (DiaDent Group International Inc., Chongju, Republic of Korea). After sampling, each paper point was placed in a sterile 1.5 mL Eppendorf tube with 100 μL sterile deionized water and stored at −70 °C before DNA extraction. The isolation of bacterial DNA was performed by 10% proteinase K (Thermo Fisher Scientific, Waltham, MA, USA) for 30 min at 56°; its inactivation was carried out by heating for 15 min at 94 °C.

Bacterial DNA was isolated by treating the samples with 10% proteinase K at 56 °C for 30 min, followed by inactivation of the enzyme by heating the samples at 94 °C for 15 min in thermoshaker TS 100C (Biosan, Riga, Latvia). Twenty-five microliters of aqueous mixture containing 2.5 μL of PCR buffer, 2.5 mM MgCl2, 0.2 mmol/L dNTPs, 0.2 μM of species-specific primers, 0.1U of DreamTaq DNA polymerase (Thermo Fisher Scientific™; Waltham, MA, USA), and 4 μL of bacterial DNA sample were used for the procedure. PCR was performed in a thermal cycler (Peqlab PeqSTAR 2X; Erlangen, Germany) under the following conditions: initial denaturation (95 °C for 3 min), cycling (35 rounds of denaturation (94 °C for 45 s), hybridization (annealing temperature for each pair of primers for 60 s for each bacterium was given in Table 1), and elongation (72 °C for one minute), and final elongation (72 °C for 5 min). PCR products were separated by 8% polyacrylamide gel electrophoresis, stained with ethidium bromide, and finally visualized and photographed after exposure to a UV light transilluminator (Benchtop UV Transilluminators; UVP, LLC, Upland, CA, USA).

### 2.9. Statistical Analysis

The parameters were analyzed by the statistical program SPSS, version 15.0 (Lead Technology Inc., Charlotte, NC, USA) as mean values (X), standard deviations (SD), and medians (Me), whereby *p* < 0.05 was considered significant. The Pearson χ^2^ test (Mantel–Haenszel) for comparing proportions of categorical variables between the groups was used; the continuous variables were compared by Student’s *t*-test or Mann–Whitney test in the case of independent samples, and a paired samples *t*-test or a Wilcoxon signed-rank test was used in case of dependent samples.

## 3. Results

### 3.1. High-Performance Liquid Chromatography (HPLC) of Tinctura Paradentoica^®^ Evaluation

According to our results (Table 2), the polyphenolic composition of the Tinctura paradentoica^®^ indicates a rich and diverse profile of bioactive compounds, with notable quantitative differences among them. Rosmarinic acid was the most abundant compound, at a concentration of 1102.79 ± 21.56 µg/mL. Among the identified flavonoids, luteolin-7-*O*-glucoside was present in a relatively high concentration, indicating its significant contribution to the overall flavonoid content. Our study also revealed a notable amount of isorhamnetin and its glycosylated derivative, narcissoside, suggesting a substantial contribution of methylated flavonols to the tincture’s potential relevance to oral health.

### 3.2. Data Analysis

**Figure 1:**
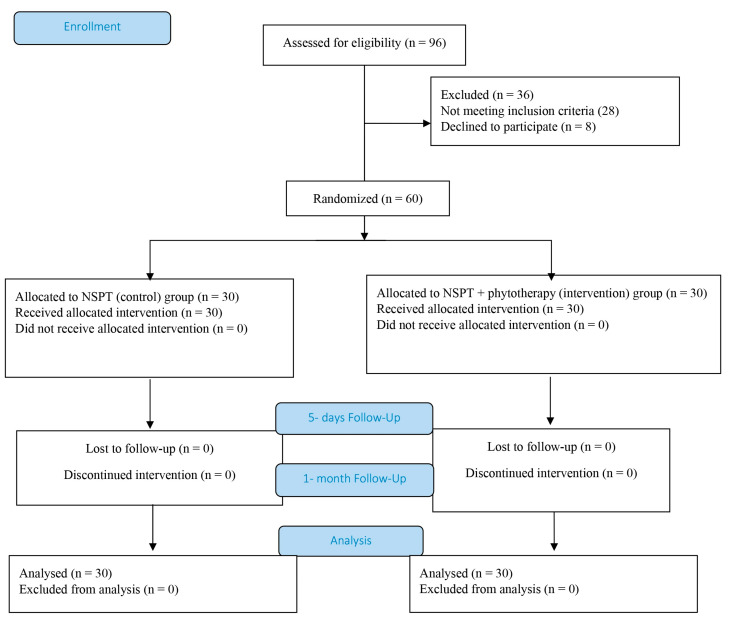
CONSORT flowchart.

A total of 96 non-smoking patients diagnosed with moderate periodontitis (Stage II, Grade A) were initially screened at the Department of Periodontology and Oral Medicine, Faculty of Medicine, University of Niš. Following the exclusion of 28 individuals who did not meet the eligibility criteria and 8 who declined participation, 60 participants were enrolled in this prospective, randomized study (Figure 1). No adverse events or harms were reported by participants during the study period.

Participants were randomly allocated to two study groups: the control group received NSPT, while the intervention group underwent NSPT supplemented with a plant-derived tincture. Demographic variables, including age and sex, did not differ significantly between the groups (Pearson’s χ^2^ test, *p* > 0.05) (Table 3).

### 3.3. Clinical Evaluation

Both groups demonstrated significant reductions in the Green–Vermilion plaque index following five consecutive treatment sessions (Wilcoxon signed-rank test, *p* < 0.001). However, 30 days after the intervention, plaque levels remained significantly elevated in the NSPT group compared to the intervention group (Mann–Whitney U test, *p* < 0.05). Similarly, the BOP index showed statistically significant reductions in the intervention group after the fifth session (*p* < 0.01), as well as at the one-month follow-up (*p* < 0.05) compared to the NSPT group. CAL improved significantly in both groups after 30 days (Wilcoxon signed-rank test, *p* < 0.001). Nonetheless, the intervention group exhibited a significantly greater gain in CAL than the NSPT group but without having CAL clinical relevance because the very small CAL difference (0.06 mm) observed between the groups. (Mann–Whitney U test, *p* < 0.001; Table 4).

### 3.4. Microbiological Evaluation

Baseline microbial analysis revealed a significantly higher prevalence of *Tannerella forsythensis* (*p* < 0.01) and *Porphyromonas gingivalis* (*p* < 0.001) in the intervention group. Post-treatment, a statistically significant reduction in *Tannerella forsythensis* and *Treponema denticola* was observed in the intervention group (*p* < 0.001), while the presence of *Porhyromonas gingivalis* remained largely unchanged (Table 5).

## 4. Discussion

Due to its multifactorial etiology and complex disease process, the treatment of periodontitis is still challenging for the dentist and dental hygienist. The benefits of this multidisciplinary approach achieved by a synergy of pharmacy and dentistry improved the periodontal care and outcomes through the development of a treatment plan.

In this study, phytotherapy, when combined with NSPT, was demonstrated to be effective in reducing periodontal clinical parameters and the prevalence of the following bacteria species (*Tannerella forsythensis* and *Treponema denticola*) in patients with moderate periodontitis.

The traditional approach to managing periodontitis has typically relied on mechanical debridement techniques, such as NSPT-scaling and root planning, and the systemic administration of antibiotics [26]. However, these methods have their limitations, as they are operator-dependent and can only temporarily control the disease, with the risk of reactivation. Moreover, the use of antibiotics has raised concerns about the development of antimicrobial resistance. Accordingly, there has been a growing interest in exploring alternative periodontal treatment approaches, including pharmacologically active phytochemicals, which involves using of tannins, terpenoids, flavonoids, and alkaloids [21]. Plant-based compounds used in the treatment of periodontitis demonstrate antibacterial, anti-inflammatory, and antioxidant properties, and also influence the structure of the periodontium [27].

All plant species that are the components of Tinctura paradentoica^®^ are known as a rich source of phenolic compounds [28,29]. The most abundant phenolic compounds in the tincture were rosmarinic acid, followed by luteolin-7-*O*-glucoside, narcissoside, and chlorogenic acid. Rosmarinic acid has shown positive effects in periodontitis in various animal and human models. Van Dyke et al. [30] used a skin graft macaque model and showed that rosmarinic acid was able to decrease gingival inflammation as well as plaque buildup. Kostić et al. [31] used *Salvia sclarea* ethanolic extract (rosmarinic acid was predominant compound) on the lipopolysaccharide-induced periodontitis in rats and a markedly reduced infiltration of inflammatory cells and an increased presence of fibroblasts were observed. Moreover, Zdarilova et al. [32] indicated that rosmarinic acid can slow periodontitis progression by reducing the production of oxidative mediators and the inflammatory response in gingival fibroblasts. Due to its anti-inflammatory potential, luteolin was also effective in animal models to combat periodontal disease and restore damaged bone tissue [33]. Furthermore, Quan et al. [34] demonstrated that luteolin promotes osteogenic differentiation in human periodontal ligament cells by activating the Wnt/β-catenin signaling pathway. Both, in vitro and in vivo studies pointed out that luteolins’ anti-inflammatory activity is connected with the inhibition of different pro-inflammatory cytokines, such as TNF-α, and modulation of the NF-κB pathway [35]. Moreover, luteolin showed great antimicrobial activity against oral microbes such as *Porphyromonas gingivalis* [36]. Dubey and Dubey [37] reported antiviral activity of glycosyloxyflavone narcissoside using molecular docking studies.

Researchers revealed that, at the end of a month, the NSPT and the intervention groups showed statistically significant improvements in PI, BOP, and CAL index. This can be primarily related to the NSPT (scaling root planning therapy) provided to both groups. NSPT is considered the gold standard of periodontal therapy. This therapy effectively demonstrated large-scale restructuring of subgingival biofilm and led to improvement in clinical parameters [38,39]. Still, a month after therapy initiation, significantly decreases in the values of the PI (*p* < 0.05), BOP (*p* < 0.05) and CAL (*p* < 0.001) indices were reported in the intervention (Tinctura paradentoica^®^) group. All participants had Stage II, Grade A periodontitis, where baseline destruction is moderate and large intergroup differences in CAL are not expected over a short-term follow-up. Although examiner calibration demonstrated acceptable reproducibility within a tolerance of ≤1 mm in 96% of recordings, this level of precision does not allow for confirmation of the clinical relevance of the very small CAL difference (0.06 mm) observed between the groups of patients with Stage II, Grade A periodontitis, as such differences require calibration with duplicate measurements differing by <0.03 mm.

Herrera et al. demonstrated improvements in CAL in short-term studies, without any associated side effects from locally delivered antimicrobials in the treatment of periodontitis [40]. Pistorius et al. described a significant decrease in the gingival index values in patients who used daily herbal mouthwash containing extracts of the following plant species: *Salvia officinalis*, *Mentha piperita*, *Matricaria chamomilla*, *Commiphora myrrha*, *Carum carvi*, *Eugenia caryophyllus*, and *Echinacea purpurea* [41]. They concluded that its daily use as an additional therapy for periodontitis reduced gingival inflammation, which was in accordance with the results of this study.

Soukoulis and Hirsch reported the efficacy of a gel with tea tree essential oil (*Melaleuca alternifolia*) in controlling dental plaque biofilm, as well as a significant reduction in the BOP values in patients with chronic gingivitis [42]. Aljuboori et al. demonstrated that *Salvia officinalis* gel exhibits anti-inflammatory properties in the therapeutic management of periodontitis, as reflected by significant modulation of both clinical outcomes and immunological biomarkers measured at 7 days and 1 month following treatment [43]. Hrishi et al. described a greater reduction in gingival inflammation and improved periodontal parameters (4 weeks after) in their randomized control pilot study with green tea dentifrice [25].

Regarding microbiological observation, the prevalence of *Tannerella forsythensis* and *Porphyromonas gingivalis* in the intervention group was significantly higher before the study. That could be explained by many variations in the genotype distribution, due to the ethnic and geographical origin of the respondents, as well as the changes in the microbiological structure of subgingival dental plaque in correlation with the individual oral hygiene method, specific lifestyles, eating, consuming alcohol, tobacco, etc. [44].

Researchers have reported that the rate of prevalence of periodontopathogenic microorganisms (*Treponema denticola* and *Tannerella forsythensis*) was significantly decreased after the phytotherapy. Still, no statistically significant difference was found for *Porphyromonas gingivalis*. These results are often contradictory because the high prevalence of the *Porphyromonas gingivalis* in healthy sites and non-periodontitis subjects has been previously reported in other populations. Some specific genotypes of this microorganism were associated with different stages of periodontitis [45,46]. Günther et al. [47] demonstrate that the tested *Rosmarinus officinalis* extract, characterized by a high concentration of phytochemicals, including rosmarinic and chlorogenic acids, exhibits a pronounced antimicrobial activity against bacteria in the early stages (2-hour-old) of oral biofilm formation, indicating its strong potential for the prevention and management of oral conditions such as dental caries and periodontitis.

Different studies have used different methods for sampling bacteria (saliva, dental plaque, mucous membranes of the oral cavity, subgingival region) and different methods of analysis (cell culture, dark-field microscopy, real-time PCR, or multiplex PCR), which significantly complicates the comparison of the obtained results [46,47,48,49].

It should be pointed out as a limitation of the study that the PCR technique used in this research did not perform a quantitative but only a qualitative analysis of the periodontal pathogens, and therefore could not detect the exact number of reduced microorganisms. Quantitative PCR analysis would undoubtedly help in determining the impact of different types of therapies on the number of periodontpathogenic bacteria [49,50]. The lack of prospective trial registration, which, although not mandatory at the time of initiation, is recommended by ICMJE (International Committee of Medical Journal Editors) and CONSORT (Consolidated Standards of Reporting Trials) guidelines for randomized controlled trials, is also a limitation of this study. Nevertheless, this does not affect the validity or reliability of the study findings (Table S1) [51].

An increase in phytotherapy and plant-derived medications, like the additional treatment in modern management of periodontitis over classic antibiotic therapy, was found [52]. Bacterial resistance is becoming a growing concern in modern medicine. Recent research in ethnopharmacology has highlighted that medicinal plants serve as valuable sources of antibacterial agents. The synergistic interaction between herbal extracts and established antibiotics can amplify this potential. Abullais Saquib et al. demonstrate that synergy tests indicated notable antibacterial enhancement when plant extracts were used alongside antibiotics [53]. Among the tested combinations, *Punica granatum* (pericarp) and amoxicillin showed the most potent synergistic effect against *Aggregatibacter aactinomycetemcomitans*, followed by *Azadirachta indica* (bark) with tetracycline [53]. These synergistic effects could aid in refining treatment approaches for periodontal infections.

Phytotherapy, in addition to its therapeutic effects, increases the general immune response, and natural products, including isolated phytochemical extracts and polyherbal formulations, have emerged as efficacious and well-tolerated therapeutic agents in the management of gingivitis and periodontal disease [54,55].

Plant-derived natural bioactive compounds make them an attractive remedy for many oral pathogens [56]. To help control the progression of periodontitis, the clinician must be familiar with phytotherapy with no relevant adverse effects. In addition, proper plant-based therapy must be based on the subject’s individual characteristics [57].

Although natural products—such as isolated plant extracts and polyherbal formulations—have demonstrated encouraging results in the management of moderate periodontitis, the current body of evidence is constrained by the absence of standardized randomized controlled trials (RCTs) and a lack of long-term clinical follow-up. These limitations underscore the necessity for well-designed, large-scale, and longitudinal studies to validate the efficacy, safety, and clinical applicability of phytotherapeutic approaches in periodontal therapy.

## 5. Conclusions

The findings of this randomized prospective study suggest that adjunctive use of an herbal tincture with conventional NSPT yields enhanced short-term outcomes in patients with moderate periodontitis (Stage II, Grade A). The intervention group experienced superior improvements in plaque accumulation and bleeding on probing, along with notable reductions in specific periodontopathogens (*Tannerella forsythia* and *Treponema denticola*). These results underscore the potential of phytotherapy as a supportive modality in periodontal management. Further large-scale and long-term studies are warranted to validate these findings and establish standardized clinical protocols for phytotherapeutic applications.

## Figures and Tables

**Table 1:** Primer sequences and PCR conditions for the amplification of bacterial DNA.

Bacteria	Primer	Base Pairs in DNA	Annealing Temperature
*Porphyromonas gingivalis* (*Pg*)	PGF PGR	5′-AGGCAGCTTGCCATACTGCG -3′ 5′-ACTGTTAGCAACTACCGATGT-3′	55 °C
*Tannerella forsythensis* (*Tf*)	TanF TanR	5′-GCGTATGTAACCTGCCCGCA-3′ 5′-TGCTTCAGTGTCAGTTATACCT-3′	55 °C
*Treponema denticola* (*Td*)	TDF TDR	5′-TAATACCGAATGTGCTCATTTACAT-3′ 5′-TCAAAGAAGCATTCCCTCTTCTTCTTA-3′	60 °C

**Table 2:** The content of individual polyphenolic compounds in Tinctura paradentoica^®^.

Compound	Content (µg/mL)
Chlorogenic acid	162.85 ± 4.12
Rutin	52.59 ± 1.39
Gallic acid	11.12 ± 0.23
Rosmarinic acid	1102.79 ± 21.56
Isorhamnetin	24.17 ± 0.49
Narcissoside	227.34 ± 3.78
Vitexin	73.17 ± 2.15
Luteolin-7-O-glucoside	358.06 ± 5.64
Hyperoside	19.89 ± 0.33

**Table 3:** Gender and age-related differences.

	NSPT (Control) Group	Intervention Group
Gender (man/woman)	11/19	11/19
Mean age	42.03 ± 15.39 (38.50)	45.03 ± 16.13 (39.00)

Results are expressed as mean ± standard deviation (median).

**Table 4:** Green–Vermilion plaque index (PI), bleeding on probing (BOP) before, after the 5 day-session treatments, and the 30 days following therapy initiation; clinical attachment level (CAL) before and 30 days following therapy initiation.

Index	NSPT Group	Intervention Group
PI Before	1.92 ± 0.56 ^#†^***(2.00)	1.93 ± 0.68 ^#†^***(2.00)
After 5th	0.00 ± 0.00 (0.00)	0.00 ± 0.00 (0.00)
After a month	1.01 ± 0.49 ^$^* (1.00)	0.66 ± 0.33 (0.70)
BOP Before	1.33 ± 0.48 ^#†^*** (1.00)	1.33 ± 0.48 ^#†^*** (1.00)
After 5th	0.52 ± 0.50 ^$^** (0.75)	0.23 ± 0.50 (0.00)
After a month	0.58 ± 0.66 ^$^* (0.20)	0.37 ± 0.50 (0.00)
CAL Before	2.89 ± 0.35 ^†^*** (3.00)	2.80 ± 0.31 ^†^*** (3.00)
After a month	2.37 ± 0.35 ^$^*** (2.42)	2.22 ± 0.26 (2.17)

Results are represented as X ± SD (Me); ^#^—vs. after the 5-day session treatments, ^†^—vs. the 30 days following therapy initiation (Wilcoxon signed-rank test). ^$^—vs. intervention group, (Mann–Whitney test). *—*p* < 0.05, **—*p* < 0.01, ***—*p* < 0.001.

**Table 5:** PCR-based identification of “red complex” bacteria before and after the 5-day session treatments.

Bacteria		NSPT Group	Intervention Group
*Tf*	Before	50.00% (15/30)	80.00% (24/30) ^a^**^C^***
	After	53.33% (16/30)	33.33% (10/30)
*Td*	Before	40.00% (12/30)	60.00% (18/30) ^C^***
	After	40.00% (12/30)	16.67% (5/30)
*Pg*	Before	50.00% (15/30)	90.00% (27/30) ^a^***
	After	43.33% (13/30)	86.67% (26/30)

Results are expressed as % (positive results/number of samples); *Tf*—*Tannerella forsythensis*, *Td*—*Treponema denticola*, *Pg*—*Porphyromonas gingivalis*. ^C^—vs. after treatments, ^a^—vs. NSPT group, **—*p* < 0.01, ***—*p* < 0.001 (Mantel–Haenszel test).

## Data Availability

The original contributions presented in this study are included in the article/Supplementary Materials. Further inquiries can be directed to the corresponding authors.

## Supplementary Materials

The following supporting information can be downloaded at: https://www.mdpi.com/article/10.3390/pathogens14090861/s1, Table S1: CONSORT 2025 checklist [51].

## Abbreviations

The following abbreviations are used in this manuscript:

NSPT	Non-surgical periodontal therapy
PCR	Polymerase chain reaction
CAL	Clinical attachment level
BOP	Bleeding on probing
PI	Green–Vermilion plaque index
*Pg*	*Porphyromonas gingivalis*
*Tf*	*Tannerella forsythensis*
*Td*	*Treponema denticola*
ICMJE	International Committee of Medical Journal Editors
CONSORT	Consolidated Standards of Reporting Trials
